# Sick of Robots—Heterogeneous Effects of Industrial Robots on Sickness Absence

**DOI:** 10.1002/hec.70010

**Published:** 2025-07-07

**Authors:** Janis Umblijs, Kjersti Misje Østbakken

**Affiliations:** ^1^ Institute for Social Research Oslo Norway; ^2^ Norwegian Social Research (NOVA) Oslo Metropolitan University Oslo Norway

**Keywords:** firm‐level analysis, industrial robots, manufacturing, sickness absence

## Abstract

This paper studies how the introduction of industrial robots affects sickness absence among workers in the manufacturing sector in Norway. We use data on the imports of industrial robots at the firm level, combined with employee‐firm linked register data, to investigate the impact of robotization on the duration of sick leave (SL). We find that robotization leads to a statistically significant increase in SL duration of approximately 1.7 days. Workers in blue‐collar occupations are especially negatively affected, and among this group those with routine tasks experience even higher levels of SL following robotization, with an average increase of around 5 days. We conduct additional analyses looking at different categories of diagnoses across various occupation groups and find heterogeneous effects. Our results suggest that for blue‐collar and routine workers robotization leads to increased musculoskeletal SL, while we only observe an increase in injuries for STEM workers, with maintenance engineers especially negatively affected. Our findings suggest several mechanisms that differ by type of occupation, ranging from musculoskeletal diagnoses caused by repetitive strain to an increase in injuries resulting from working directly with the newly installed industrial robots.

## 1 Introduction

Industrial robots have significantly impacted the manufacturing sector in recent decades, with the International Federation of Robotics (IFR) citing an annual growth of 14% over the next 3 years (IFR 2023). Recent research provides cause for concern that the widespread automation of tasks that previously were performed by workers, can have a disruptive effect in terms of displacement and real wage stagnation for some groups of workers (see, e.g., Acemoglu et al. 2023; Acemoglu and Restrepo 2020; Barth et al. 2025; Bessen et al. 2023; Dauth et al. 2021). In this paper, we add to the current state of knowledge on labor market effects of robots by incorporating the worker health perspective. Using data on the imports of industrial robots at the firm level, combined with employee‐firm linked register data, we study how robotization impacts workers' sick leave (SL), which can be costly for firms and negatively impact workers. We apply a stacked difference‐in‐differences event‐study methodology to estimate the effects of robotization on SL for 4 years post‐automation, using firms that robotized later as a control group.^1^ The data includes detailed information on the incidence, duration and diagnoses of doctor‐certified SL at the individual level. We supplement this individual‐level study with a firm‐level analysis to investigate the role that worker composition plays in the link between robotization and SL.

Our findings indicate a statistically significant increase of around 1.7 SL days among workers who stay in the firm at least 3 years prior to and 4 years after robotization. Additionally, we find that *blue‐collar* workers and those working in occupations with routine tasks are especially negatively affected, while we find no SL effects for *office* workers. Looking at different diagnosis categories, our findings suggest that while blue‐collar workers have the highest increase in SL days due to musculoskeletal diagnoses, it is only for *STEM* workers, which includes industrial robot maintenance workers, that we observe an increase in SL due to injuries. The firm‐level analysis finds a larger SL effect due to sicker workers being more likely to stay in the firm following robotization.

This paper contributes to the rapidly expanding literature on automation and the labor market using individual‐level data. These studies have generally found negative effect on employment and wages for blue‐collar workers (Acemoglu et al. 2023; Barth et al. 2025; Bessen et al. 2023; Bonfiglioli et al. 2022; Humlum 2021; Krenz et al. 2021), while results differ for other groups of workers. Given these negative employment and wage effects, one might expect robotization to be linked with health outcomes as well. On the one hand, the introduction of industrial robots may create safer work environments and prevent illness, injuries and accidents resulting from dangerous work. On the other hand, technological upgrading creates new health risks. Specifically, workers who are responsible for installing, running and maintaining industrial robots may be at a higher risk of injury (Lee et al. 2021; Yang et al. 2022), while those working downstream from robots in the production process may experience health issues due to increased repetitiveness of new tasks or a higher pace of production. Robotization can also lead to job insecurity, which could increase mental health hazards (Du et al. 2023; O’Brien et al. 2022). Additionally, if robotization is perceived as a threat to job security, the introduction of industrial robots could discourage workers from taking sick leave as it may increase the risk of displacement (Allen 1981; Dunn and Youngblood 1986; Johansson and Palme 2005). On the other hand, if robotization is a controversial decision, it could prompt a retaliatory behavior where workers use sick leave as a form of “punishment” toward employers (Fehr and Gächter 2000).

The existing empirical literature does not point to a clear expectation of how industrial robots might affect workers' sickness absence. On the one hand, several studies find evidence that automation improves worker health. Using data from the IFR to predict regional robot penetration in the US, Gunadi and Ryu (2021) find that robotization is associated with an improvement in self‐reported health among low‐skilled workers living in a region with a high estimated level of robotization. Gihleb et al. (2022) corroborate this finding with data on doctor‐assessed disability and find that robotization was linked with a decline in disability and reduction in jobs with physically intensive tasks in Germany. Both articles suggest that these health benefits arise from robotization leading to a shift away from physically demanding and dangerous tasks. Other studies provide evidence for this mechanism with findings that certain types of industrial robots improve workplace safety by taking over dangerous, strenuous, or repetitive tasks done by workers (Kim et al. 2017). None of these studies have information on both SL at the individual level and robotization at the firm level, making it difficult to conclude regarding direct health effects for those working in firms that have been robotized.

A growing literature also shows that the ongoing process of automation leads to expectations of increasing job insecurity, which, in turn, may affect health negatively (De Witte et al. 2016; Khubchandani and Price 2017; Patel et al. 2018; Reichert and Tauchmann 2017). More specifically, that higher robot intensity is negatively associated with self‐reported mental health, due to concerns over job security and dissatisfaction with task changes post robotization (Abeliansky and Beulmann 2019). Research from Norway using national‐level data on robotization form the IFR suggests that the fear of introducing industrial robots may negatively affect job satisfaction, especially for workers carrying out routine tasks (Schwabe and Castellacci 2020). In a similar vein, using European‐wide data, Gorny and Woodard (2020) find that working in occupations with a higher risk of automation is associated with a decrease in job satisfaction. Using survey data from Taiwan, Cheng et al. (2021) show that occupations with high risk of automation experience lower job control, higher job insecurity and higher risk of work‐related injuries and diseases. Research from the US has found that automation, measured at the regional level, may be linked to increases in drug overdoses (Venkataramani et al. 2020) and suicide (O’Brien et al. 2022). Evidence from China also suggest that robot exposure leads to increased alcohol consumption and problem drinking (Lu and Fan 2024). The broader literature on the effect of job insecurity and declining economic opportunities on sickness absence is also inconclusive. Several studies show that SL is lower in less secure jobs (Arai and Thoursie 2005; Ichino and Riphahn 2005; Johansson and Palme 2002), while others find increasing sickness absence from downsizing (Røed and Fevang 2007) and a decrease in SL from a financial negative shock at the municipal level (Bratberg and Monstad 2015).

This paper contributes to the existing literature in several ways. First, it is the first to analyze the effects of robotization on health using data that combines the introduction of industrial robots at the firm level with individual‐level health data. By not relying on national industry‐level data for robotization but with a direct link between robotizing firms and the individual, we are able to study the impacts on SL for directly affected workers. A second major contribution of the paper is the use of detailed data on SL, including information on the medically certified diagnosis. This allows us to disaggregate SL caused by musculoskeletal, psychological and injury diagnoses. Finally, our data contain detailed occupation codes, which we use to test for heterogeneity of effects for different categories of workers. The remainder of the paper is structured as follows: section two outlines the institutional background relevant for SL in Norway, section three introduces our data, section four describes our empirical strategy, section five presents our individual‐ and firm‐level results and section six concludes.

## 2 Institutional Background

Norway's extensive SL system covers employees who have been employed at the same workplace for at least 4 weeks, offering mandatory, law‐regulated 100% sickness insurance coverage for up to 1 year.^2^ Employers pay for the first 16 days of sickness, after which the Norwegian Labor and Welfare Administration (NAV) takes over. If sickness exceeds three or 8 days, depending on whether employer's adherence to the Tripartite Agreement of a more inclusive working life, a medical certificate is needed.

A prevalent view is that sickness absence levels are too high and expensive, partly due to the generosity of the system (Hemmings and Prinz 2020), sparking concern that the transition out of the workforce starts with sickness absence. Proposals to curb sickness absence, such as reducing the replacement ratio or to increase the employer period are highly controversial and have not yet been implemented. Instead, other efforts to reduce sickness absence have been introduced such as the Tripartite Agreement of a more inclusive working life (2001) aiming to reduce the level of sickness absence by 20% through improving working conditions and providing better SL follow‐up. Changes were also made to doctors' certification rules in 2003. Despite multiple renegotiations and stronger early‐phase efforts, more use of graded absences and dialog meetings, and a more work‐oriented system for health‐related benefits, success in reducing sickness absence has been limited.

Other aspects of worker protection are relatively strong in Norway, protecting employees from unfair dismissals based on factors such as age, trade union activities, military service, pregnancy and recent motherhood and sickness absence (OECD 2020). Furthermore, union membership is prevalent, with over half of the workforce being part of a union. High rates of collective bargaining and a strong tradition of cooperation between the state, unions, and employer associations on matters concerning employment, wages and the welfare benefit system are also key features of the Norwegian labor market.

## 3 Data

We employ linked employer‐employee administrative register data from Statistics Norway for the period 2003–2018. These datasets are linked to firm‐level information on industrial robot imports in the manufacturing sector, register data on medically certified sickness absence at the individual level and individual registers containing educational and demographic information.

We identify robotizing firms as those that have imported an industrial robot based on the Norwegian Trade Statistics Register.^3^ Norway's domestic production of industrial robots is primarily focused on oil and gas and underwater technologies. Therefore, most manufacturing firms outside of these sectors import industrial robots from abroad. Furthermore, we exclude firms in wholesale sectors to avoid capturing importers of robots that sell them on to others.

To check how well our import measure reflects robotization in the Norwegian manufacturing sector, we compare the imports of robots with data from the IFR, which collects yearly data from members on the new installation of industrial robots.

**FIGURE 1:**
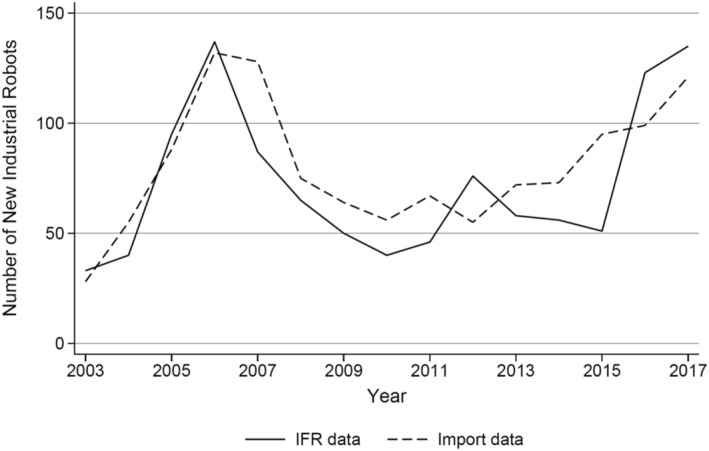
Import measure of robotization and data from the IFR. The IFR data show the yearly number of new installations of industrial robots in the manufacturing sector based on a survey from its members in Norway. The import data comprise the total number of robots imported by manufacturing firms as identified by their NACE industry codes.

Figure 1 shows that for the manufacturing sector the yearly imports of robots closely match the data from the IFR, suggesting that the import measure approach we apply is well suited for identifying robotizing firms, while allowing us to identify these firms and link them to those employed there.

**FIGURE 2:**
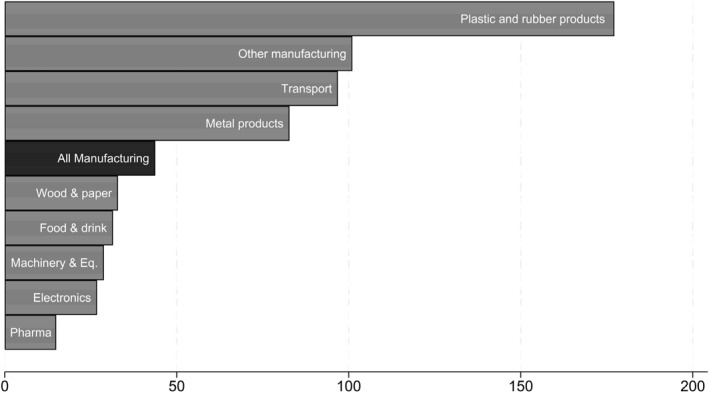
Robot penetration by sector: number of robots per 10,000 workers. The figure shows the extent of robot penetration by sector. It shows the operational stock of industrial robots (source: IFR statistics) per 10,000 employees in each industry as well as the average for the whole manufacturing industry (source: Norwegian register data). The categories are 2‐digit NACE codes (plastic and rubber = 22; other manufacturing = 23, 31, 32; transport = 29, 30; metal products = 24, 25; wood and paper products = 16, 17, 18; food and drink = 10, 11, 12; machinery and equipment = 28; computers and electronic equipment = 26, 27; chemicals and pharmaceuticals = 19, 20, 21).

**FIGURE A2:**
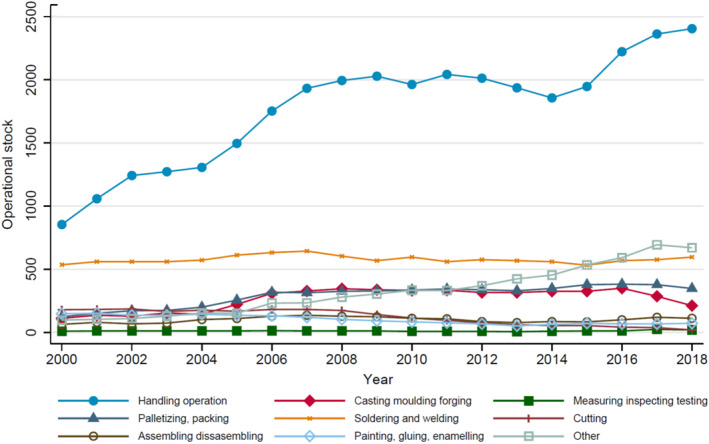
Operational stock of industrial robots in Norway by application. The figure shows the installation of industrial robots by robot application in the manufacturing sector in Norway. The data are from the International Federation of Robotics (IFR) and are based on survey responses from members of the organization in Norway.

Compared to other European countries the number of industrial robots per worker in Norway is relatively low, with 44 industrial robots per 10,000 employees in the manufacturing sector, compared to an average of 136 in Europe (IFR 2024).^4^ However, robot penetration for specific industries is somewhat higher (See Figure 2).^5^ The results of this paper are therefore most directly relevant for countries with similar patterns of robot adoption as well as specific industries where Norway has higher levels of robot adoption. Regarding robot applications, data from the IFR suggest that, like in most other countries, handling robots are by far the most common category (see Figure A2 in Appendix C).

Using robot import data, we create a binary indicator assigning 1 to the first year a firm imports an industrial robot and 0 otherwise. We then track individuals in these firms for 4 years post‐robot introduction, assuming a robot lifespan of 6–7 years, as shown in the existing literature (Karastoyanov and Karastanev 2018). Information on medically certified SL is collected from the sickness absence register. These data comprise a wide range of information on individual‐level physician‐certified SL, such as the start and end dates of absence spells, whether sickness absences are full‐ or part‐time and the ICPC‐2 diagnosis of each spell. All physician‐certified sickness absence spells are reported to the NAV in Norway. The sickness absence register is therefore a reliable source of all SL spells that have been assessed and approved by medical professionals. Our key dependent variable is SL days at the individual level within a calendar year.

In the analysis, we investigate the effect of robotization on the total number of SL days, on SL days with the two largest diagnosis groups separately (musculoskeletal and psychological), and injury related SL. We create these diagnosis groups using the ICPC‐2 classification (see Appendix A, Table A1). Note that the musculoskeletal and psychological diagnosis categories are mutually exclusive—an SL spell can only be registered under one diagnosis, while injuries comprise diagnosis codes from across the ICPC‐2 classification.

**FIGURE 3:**
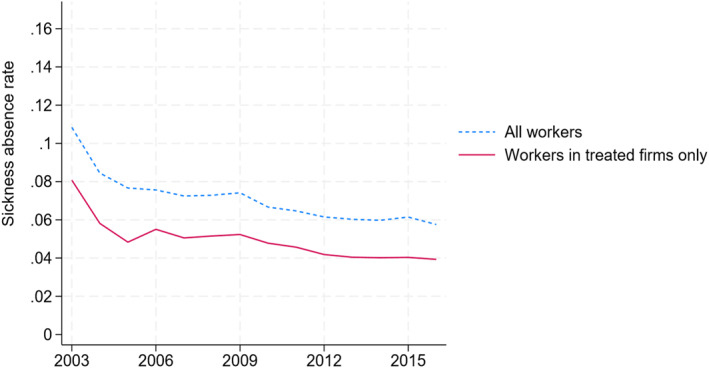
Sickness absence rates in Norway (2003–2018). All workers and workers in treated firms only. The sickness absence rate is a weighted sum of the medical certified days of SL relative to the weighted days worked. The weights on absences are the sickness absence grade, while the weights on days worked are the share of a full‐time position.

**FIGURE 4:**
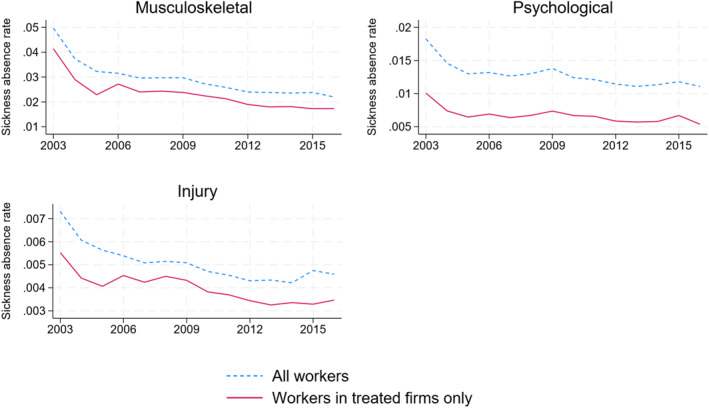
Sickness absence rates in Norway (2003–2018). All workers and workers in treated firms only by musculoskeletal and psychological diagnoses and injuries. The sickness absence rate is a weighted sum of the medical certified days of SL relative to the weighted days worked. The weights on absences are the sickness absence grade, while the weights on days worked are the share of a full‐time position. The diagnosis groups are based on the first digit in the ICPC‐2 classification: musculoskeletal (L), psychological (P). Injuries include all diagnosis codes that are classified as injuries (see Appendix A).

As shown in Figure 3, the sickness absence rate in Norway has been declining in our observation period, in particular after 2003 when new regulations on doctors' certification practices were introduced. Since 2010, the absence rate has been stable at around 6% among all employees. The sickness absence rates among the workers in our sample of treated firms follow a similar pattern but are slightly lower overall. Figure 4 shows that the sickness absence rate is highest for musculoskeletal diagnoses, followed by psychological. The absence rate for injuries is low, at less than 0.5%.

As exposure to robots differs across occupational tasks, we look at the heterogeneity of effects for different types of workers. Firstly, we differentiate between the occupation categories of *office workers,* engineers and technicians (*STEM*) and *blue‐collar* workers.^6^ This three‐category classification is relevant for our analysis as it gives an indication of the proximity of the tasks in these occupations to the new technology. For example, while we expect *blue‐collar* and *STEM* workers to directly engage with the new technology, *office workers* might only be affected indirectly through the consequences of robotization on employment and wage‐setting strategies.

While robots that operate side by side with workers exist, “these types of collaborative robots remain a niche in the global industrial robot market, representing only 7.5% of the market” (Gambao 2023, 1). Most industrial robots operate in areas physically separated from other workers, and because of their weight and speed, they are usually located in a caged zone for safety reasons. Only robotics installers and maintenance engineers have direct access to these industrial robots to install, program, service and maintain them.^7^ While *blue‐collar* workers performing tasks downstream in the production process can be affected by robotization as it may change the content and speed of their tasks (Antón et al. 2023; Koppenborg et al. 2017), they are unlikely to directly interact with industrial robots.^8^

Other dimensions of occupational tasks are also relevant. Among *blue‐collar* workers we expect, based on the existing literature, that those working with simple and repetitive tasks might be especially affected by changes in the production process caused by technological upgrading (see, e.g., Abeliansky and Beulmann (2019) and Gorny and Woodard (2020)). To identify this difference, we split our sample into routine and non‐routine workers based on the occupational codes of their employment relationships.^9^

Table 1 shows the number and share of workers in the three different occupation categories in our sample of robotizing firms and whether an occupation is categorized as having predominantly routine tasks. It shows that routine workers are exclusively found among *blue‐collar* occupations, with 11% of workers in our sample categorized as this.

**TABLE 1:** Descriptive statistics of the number and share of workers in the occupation categories.

Occupation		Non‐routine	Routine	Total
Blue‐collar	*N*	191,733	50,010	241,743
	Row %	79.31	20.69	100.00
	Col %	45.32	100.00	51.10
Office worker	*N*	118,604	0	118,604
	Row %	100.00	0	100.00
	Col %	28.04	0	25.07
STEM	*N*	112,694	0	112,694
	Row %	100.00	0	100.00
	Col %	26.64	0	23.82
Total	*N*	423,031	50,010	473,041
	Row %	89.43	10.57	100.00
	Col %	100.00	100.00	100.00

*Note:* This table shows the total number and share of workers, as well as routine and non‐routine workers, in each of the three occupation categories.

Table 2 displays descriptive statistics with respect to SL across various diagnoses and several individual characteristics. In addition to all the categories of workers, the table also shows statistics separately for each of the three occupation categories (Panel A) and for both routine and non‐routine workers (Panel B). These statistics are for observations in the pre‐period, including both the treatment and control groups.

**TABLE 2:** Summary statistics of the main variables in the pre‐period.

**Panel A: All, routine and non‐routine worker categories**			
**Variables**	**All workers**	**Routine**	**Non‐routine**
SL days	11.128 (29.888)	2.265 (32.365)	110.985 (29.557)
SL categories			
Musculoskeletal	5.565 (22.417)	6.676 (25.595)	5.425 (21.978)
Psychological	1.239 (10.268)	1.211 (10.507)	1.243 (10.237)
Injury	1.236 (9.478)	1.519 (10.693)	1.200 (9.313)
Individual characteristics			
Yearly earnings	535,632 (368,234)	449,889 (141,020)	546,476 (386,242)
Age	43.214 (9.327)	42.492 (10.165)	43.036 (8.805)
Female	0.214 (0.410)	0.356 (0.479)	0.244 (0.430)
Higher education	0.265 (0.441)	0.051 (0.220)	0.292 (0.455)
Observations	197,068	222,11	174,949

**Panel B: Blue‐collar, office worker and STEM occupation categories**			
**Variables**	**Blue‐collar**	**Office worker**	**STEM**
SL days	15.120 (34.802)	7.909 (24.719)	5.381 (19.553)
SL categories			
Musculoskeletal	8.284 (27.415)	3.152 (16.461)	1.874 (11.781)
Psychological	1.369 (10.910)	1.299 (10.757)	0.885 (8.012)
Injury	1.754 (11.236)	0.683 (6.895)	0.626 (7.015)
Individual characteristics			
Yearly earnings	424,182 (129,688)	612,042 (641,380)	710,098 (246,450)
Age	42.233 (9.650)	45.578 (8.657)	43.036 (8.805)
Female	0.214 (0.410)	0.356 (0.479)	0.146 (0.353)
Higher education	0.040 (0.197)	0.379 (0.485)	0.656 (0.475)
Observations	104,177	46,682	46,209

*Note:* The above table shows the average number of SL days as well as selected individual characteristics (with standard deviations in parentheses) for all, routine and non‐routine workers (Panel A) and *blue‐collar*, *office* and *STEM* workers (Panel B). The table only includes observations from the pre‐period.

## 4 Empirical Strategy

We exploit the panel structure of the data and follow a stacked difference‐in‐differences event‐study approach, similar to among others, Bessen et al. (2023). We identify the group of treatment firms by year *c*, which is the year that a firm invested in robots. Individuals working in these firms in year *c* are then followed in the event window from 3 years before to 5 years after robotization {−3, …, 4}. The control group of firms are those that introduced industrial robots in year c + 5 or later. Individuals in these firms are followed in the same period as those in the treatment group {−3−, …, 4}. For example, treatment firms that introduced a robot in 2006 are compared to a group of control firms that did so in 2011 or later. Both groups of individuals are then evaluated in the same period, that is, 2003–2010. This exercise is then repeated for each cohort. Finally, we stack all the cohort datasets along the time window relative to robotization {−3‐, …, 4}. This gives us a balanced panel data in event time, thereby preventing problems related to the staggered timing of treatment, extensively discussed in the recent difference‐in‐differences literature (see, e.g., Sun and Abraham 2021; De Chaisemartin and D’Haultfoeuille 2023; Goodman‐Bacon 2021). We estimate variants of the following equation:

(1) $${\;Y}_{ijt}=\alpha +\beta \;{Treat}_{i}+\sum_{\tau =\neq -1;\;\tau =-3}^{4}\gamma_{\tau }I_{\tau }*{Treat}_{i}+\sum_{\tau =\neq -1;\tau =-3}^{4}\delta_{\tau }I_{\tau }+\varphi_{t}+\pi_{i,k}+\varepsilon_{jt}$$

where *Y*_*ijt*_ denotes the outcome for individual *i* working in firm *j* at time *t*, and *τ* is the event time (−3, …, 4). *Treat* equals 1 for individuals working in firms that import robots at *τ* = 0. *I*_*τ*_ are event‐time indicators, with *τ* = −1 as the reference category. The key parameters are $\gamma_{\tau }$. These coefficients identify the relative changes in the outcome variable relative to the reference event time (*τ* = −1) for individuals in robotizing firms (treat) versus firms that robotize later (control).

Furthermore, $\varphi_{t}$ denotes calendar‐year fixed effects, $\pi_{i,k}$ are the cohort by individual fixed effects and are included to account for the fact that individuals can enter both the treatment and control groups between different event cohorts. Standard errors are clustered at the firm‐cohort level. We are interested in the effect of robotization on individuals who keep working in the firm; therefore, our sample is balanced and only includes individuals observed over all *τ* periods who have remained in the same firm the whole time. The focus on those who do not change jobs is also justified by the fact that SL is linked to firms and therefore including individuals who change jobs would require the problematic assumption that sick leave at a new firm is caused by robotization at the previous firm.

The stacked difference‐in‐differences event‐study approach helps to ensure that the treatment and control groups are comparable in terms of their observable characteristics. To increase the comparability of these two groups further, we also balance treatment and control along the dimensions of wages, age, gender, education and the size of the company using entropy balancing, as developed by Hainmueller (2012). Entropy balancing improves comparability between our treatment and control groups, predominantly through weighting by wages and firm size (number of employees); it does not, however, have a substantial impact on our results. More details on the balancing procedure and a comparison of the balanced and non‐balanced estimates are provided in Appendix D.^10^

For brevity, for some of the analysis we estimate variants of (1) using the same stacked sample and year observations but only for two periods: pre‐ and post‐robotization. We include category interaction terms for occupational groups and the routine dummy to estimate heterogeneous effects. In addition to calendar year effects (as shown in Equation 1), here we also control for occupation‐group‐specific trends by including a *year × occupation category* interaction term in the estimation. To take account of the imports of industrial robots late in the event year (*τ* = 0) and the fact that setting up industrial robots takes time, we apply the “donut” estimation approach and exclude this year from our estimation of the average treatment effect in the post‐period. We do this by adding an interaction term between each group of workers and the year of robot imports. The estimated effect thus compares outcomes in the years before to the years after robot imports, with relative outcomes in the event year allowed to vary freely (see, e.g., Deshpande et al. (2021) and Propheter (2020) for an application of this approach in other contexts).

Finally, we study the impacts of robotization on SL at the firm level in order to explore the effect of worker compositional factors on the relationship between robotization and SL.

## 5 Results

**FIGURE 5:**
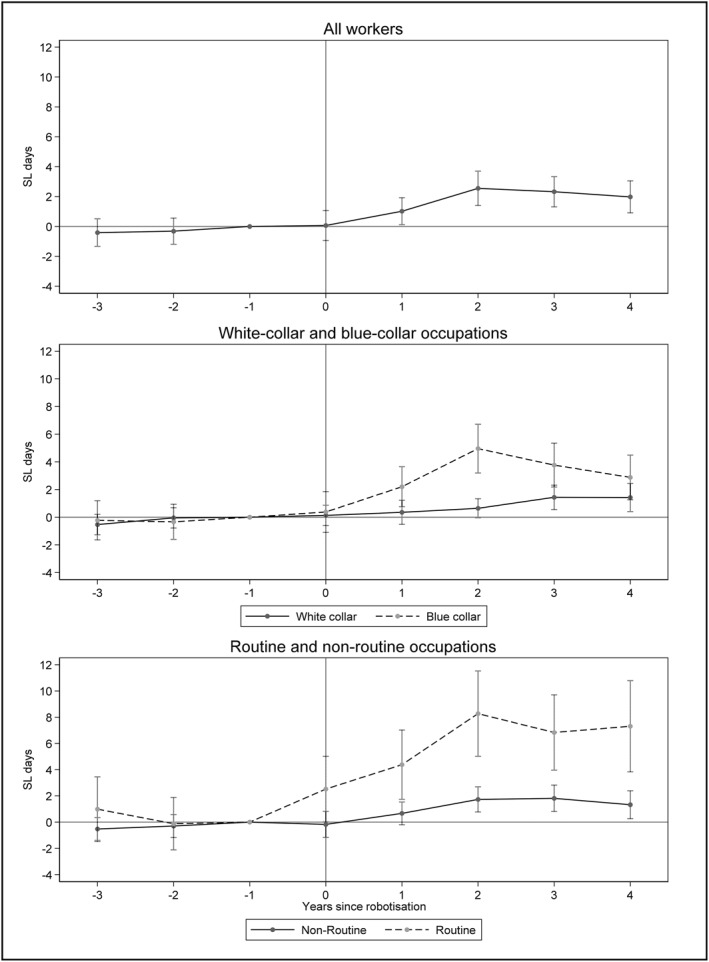
Difference‐in‐differences event study: impact of robotization on SL. The outcome variable is the total SL days, measured yearly. The sample includes individuals who worked in the same firm and were observed in all the years of the estimation period *τ* ∈ {−3, …, 4}. Standard errors are clustered at the firm level, and the sample is weighted using entropy balance weights. The whiskers reflect 95% confidence intervals.

We investigate the impact of robotization on SL by estimating a stacked difference‐in‐differences event‐time model, as described in section four. The top panel of Figure 5 shows the difference between the treated and control groups regarding deviations from the number of days of SL 1 year before the treatment year (*τ = −*1). It shows that there were no significant differences in the treatment and control groups in the pre‐period, suggesting parallel trends.^11^ Following robotization, SL days increased in the year after robotization with a statistically significant effect in the second, third and fourth years. The average effect of robotization over the 4 years following robotization is a statistically significant increase of 1.7 SL days (Table 2, Column 1). This is a 14% increase compared to the pre‐period SL.

In the middle panel of Figure 5, we apply the same approach as in the panel above but separately for *blue‐collar* and white‐collar workers.^12^ The results show a clear difference between the effect of robotization on SL for these two groups. While we find an effect of 0.7 days for white‐collar workers, robotization leads to a much larger increase in SL days for *blue‐collar* workers. For the latter, robotization increases SL by around 3 days per year in the post‐period.

We are also interested in the heterogeneity of effects between routine and non‐routine workers. In the bottom panel of Figure 5, we show the results for these two groups of workers. Our analysis shows that robotization has a greater impact on routine than non‐routine workers in terms of total SL days. For workers with routine tasks, robotization caused an average increase of around five SL days in the 4 years following robotization. In comparison, the average effect for non‐routine workers was lower, with an average increase of one SL day. Taken together, Figure 5 shows that robotization leads to an increase in SL and that this effect is larger for *blue‐collar* workers and especially for those that perform routine tasks.^13^

In the following, we investigate the effect of robotization on different categories of SL and the heterogeneity of these effects for the three occupation categories (*blue‐collar*, *office* and *STEM* workers), as well as for routine and non‐routine workers. Our results show that robotization increases SL for all the SL diagnosis categories except the psychological one (Table 3). The magnitude of the effect in terms of days is largest for musculoskeletal SL, with a 0.8‐day increase, which corresponds to around a 10% increase from the mean number of days per year for our sample of workers in the pre‐period. However, as SL due to injuries is the least common category in our sample before robotization, the effect relative to the mean is larger, with the 0.2‐day increase corresponding to around a 14% SL‐day increase from the mean.^14^

**TABLE 3:** Difference‐in‐differences: impact of robotization on SL by categories of diagnoses.

	(1)	(2)	(3)	(4)
	All	Muscle	Psychological	Injuries
Robotization	1.666^***^	0.838	0.182	0.172^*^
	(0.305)	(0.210)	(0.112)	(0.0790)
Constant	10.23^***^	5.501^***^	1.117^***^	1.330^***^
	(0.391)	(0.309)	(0.157)	(0.115)
Year effects	Yes	Yes	Yes	Yes
Entropy balanced	Yes	Yes	Yes	Yes
*R* ^2^	0.311	0.278	0.229	0.186
*N*	473,041	473,041	473,041	473,041

*Note:* Dependent variable: SL days. Column 1 shows the effect of robotization on all diagnoses of SL measured per year. Columns 2, 3 and 4 look specifically at musculoskeletal diagnoses, psychological diagnoses and SL days due to injuries, respectively. Standard errors are clustered at the firm level, and the sample is weighted using entropy balance weights. Standard errors in parentheses.

* *p* < 0.05.

**p < 0.01.

*** *p* < 0.001.

In addition to the increase in the number of SL days, we also estimate the impact of robotization on taking any SL, with an indicator variable for experiencing an SL spell in a given year as the dependent variable. The results show that taking any SL is not statistically significant for the all, musculoskeletal or psychological categories but is statistically significant for injury‐diagnosed SL (Table A4 in Appendix F). This suggests that the increase in SL days regarding musculoskeletal diagnoses following robotization is driven by an increased length of SL spells and not by an increase in SL incidence. The effect of robotization on injuries does however appear to be driven by an increase in SL incidence. As we are interested in the impact of robotization on both the incidence and duration of SL, the rest of the paper focuses on SL days as an outcome.

Table 4 presents results for the different SL diagnoses separately for each of our three occupation categories. We use *office workers* as our reference category and show both the deviation from the reference category as well as the total effect of robotization on SL for *STEM* and blue‐collar workers. The results show that from these three categories, robotization had the largest adverse effect on health for *blue‐collar* workers measured by SL days, with statistically significant effects for *musculoskeletal* diagnoses. Robotization increased SL days due to musculoskeletal diagnoses for *blue‐collar* but not for *STEM* or *office workers* (Column 2). Robotization did not, however, lead to a statistically significant increase in SL days due to psychological diagnoses for any of the four occupation categories (Column 3). Turning to SL due to injury, we find a statistically significant increase exclusively for *STEM* workers following the introduction of industrial robots (Column 4). Looking at the occupations that are most likely to experience injury following robotization, the most common are service and maintenance engineers in the *STEM* occupation category. Workers in these occupations are likely to come into regular and direct contact with industrial robots and are, therefore, at a higher risk of physical injury.

**TABLE 4:** Difference‐in‐differences: impact of robotization on SL by categories of diagnoses with occupation interactions.

	(1)	(2)	(3)	(4)
	All	Muscle	Psychological	Injuries
Office workers (ref.)	0.386	0.211	0.300	0.0730
	(0.433)	(0.243)	(0.197)	(0.116)
Deviation from ref. Group:				
Blue‐collar	2.353^***^	1.332^**^	−0.148	0.0819
	(0.591)	(0.428)	(0.238)	(0.189)
STEM	0.823	0.213	−0.165	0.204
	(0.538)	(0.320)	(0.236)	(0.162)
Total effect for subgroup:				
Blue‐collar	2.739	1.543^***^	0.151	0.155
	(0.496)	(0.362)	(0.159)	(0.145)
STEM	1.209^***^	0.425	0.134	0.277^*^
	(0.335)	(0.223)	(0.167)	(0.108)
Year effects	Yes	Yes	Yes	Yes
Year* subgroup effects	Yes	Yes	Yes	Yes
Entropy balanced	Yes	Yes	Yes	Yes
*R* ^2^	0.312	0.278	0.229	0.186
*N*	473,041	473,041	473,041	473,041

*Note:* Dependent variable: SL days. Column 1 shows the effect of robotization on all diagnoses of SL measured per year. Columns 2, 3 and 4 look specifically at musculoskeletal diagnoses, psychological diagnoses and SL days due to injuries, respectively. Standard errors are clustered at the firm level, and the sample is weighted using entropy balance weights. Standard errors in parentheses.

* *p* < 0.05.

** *p* < 0.01.

*** *p* < 0.001.

Turning now to routineness, Table 5 shows the heterogenous effect of robotization on the different SL diagnosis categories for routine and non‐routine workers. The results show that robotization increases SL more for routine workers than for non‐routine ones. For routine workers, we find that robotization is associated with an increase in SL days due to *musculoskeletal* diagnoses. This could be indicative of increased repetitive strain injuries caused by changes in task content and/or intensity for this group of workers (Antón et al. 2023; Koppenborg et al. 2017).

**TABLE 5:** Difference‐in‐differences: impact of robotization on SL by categories of diagnoses with routine‐task interactions.

	(1)	(2)	(3)	(4)
	All	Muscle	Psychological	Injuries
Non‐Routine (ref.)	1.187	0.610^**^	0.154	0.151
	(0.301)	(0.203)	(0.121)	(0.0775)
Deviation from ref. Group:				
Routine	3.659	1.835^**^	0.238	0.295
	(0.943)	(0.707)	(0.275)	(0.264)
Total effect for subgroup:				
Routine	4.846^***^	2.445^***^	0.392	0.446
	(0.929)	(0.698)	(0.256)	(0.261)
Year effects	Yes	Yes	Yes	Yes
Year* subgroup effects	Yes	Yes	Yes	Yes
Entropy balanced	Yes	Yes	Yes	Yes
*R* ^2^	0.311	0.278	0.229	0.186
*N*	473,041	473,041	473,041	473,041

*Note:* Dependent variable: SL days. Column 1 shows the effect of robotization on all diagnoses of SL measured per year. Columns 2, 3 and 4 look specifically at musculoskeletal diagnoses, psychological diagnoses and SL days due to injuries, respectively. Standard errors are clustered at the firm level, and the sample is weighted using entropy balance weights. Standard errors in parentheses.

* *p* < 0.05.

** *p* < 0.01.

*** *p* < 0.001.

### 5.1 Heterogeneity

In this section, we look at the heterogeneous effect of robotization on SL along several other dimensions. More specifically, in Table 6, we look at the heterogeneity of the effect of robotization on SL days according to the year of robotization, firm size, robot exposure, gender and union membership. We define later cohorts as firms robotizing in years after 2008, large firms as those that have more than the mean number of employees, high robot exposure as firms that importing industrial robots over the median value, and union members as individuals who were members of a union 1 year before the treatment. The reference groups are, therefore, firms robotizing before 2009, firms with less than the mean number of employees, firms with robot imports below the median value, men and non‐union workers. We estimate effect heterogeneity by including an interaction term for these four dimensions, with SL days for all diagnoses as the outcome variable.

**TABLE 6:** Difference‐in‐differences: impact of robotization on SL. Heterogeneity of effects.

	(1)	(2)	(3)	(4)	(5)	(6)
	Later cohort	Large firm	High robot exposure	Female	Union	Union (excl. office)
Reference group:	2.703^***^	1.502^**^	1.657***	1.504^***^	1.227	2.555^***^
Early cohort (1), small firms (2), low robot exposure (3), male (4), not union members (5,6)	(0.566)	(0.526)	(0.362)	(0.305)	(0.402)	(0.580)
Deviation from ref. Group for Later cohorts (1), large firms (2), high robot exposure (3), female (4), union members (5,6)	−1.405	0.261	0.0883	0.820	0.601	−0.301
	(0.623)	(0.630)	(0.601)	(0.730)	(0.516)	(0.658)
Total effect subgroup	1.298^***^	1.762^***^	1.745***	2.324^**^	1.828^***^	2.253^***^
Later cohorts (1), large firms (2), high robot exposure (3), female (4), union members (5,6)	(0.326)	(0.369)	(0.488)	(0.726)	(0.383)	(0.435)
Year effects	Yes	Yes	Yes	Yes	Yes	Yes
Year* subgroup effects	Yes	Yes	Yes	Yes	Yes	Yes
Entropy balanced	Yes	Yes	Yes	Yes	Yes	Yes
*R* ^2^	0.311	0.311	0.311	0.311	0.311	0.320
*N*	473,041	473,041	473,041	473,041	473,041	345,326

*Note:* Dependent variable: SL days. Standard errors are clustered at the firm level, and the sample is weighted using entropy balance weights. Standard errors in parentheses.

* *p* < 0.05.

** *p* < 0.01.

*** *p* < 0.001.

The results of the heterogeneity analysis show that while both early and late adopters experience positive and significant effects on SL, the effect is significantly lower among late adopters. The lower treatment effect among late adopters may suggest that later robots are safer for workers than earlier robots. Model 2 shows that smaller firms are somewhat more negatively affected by robotization, although this difference is small and not statistically significant. Column 3 shows that firms spending more on industrial robots experienced a larger effect of robotization on SL, but the estimate is not statistically significant.^15^ Previous research has shown that robotization has gendered impacts (Aksoy et al. 2021; Ge and Zhou 2020). Our results also show that women are more negatively affected by robotization than men, but the difference is not statistically significant (Column 4). Model 5 shows that being a member of a union is linked to a higher SL effect compared to non‐unionized workers. However, the result in column (5) is likely to be because *blue‐collar* and *STEM* workers have higher rates of unionization than *office workers* and, at the same time, are more negatively affected by robotization.^16^ Indeed, when we exclude *office workers* (Column 6), we find that being unionized reduces the negative effect of robotization on SL. We only find statistically significant differences between groups for late compared to early cohorts. There is no statistically significant difference in the treatment effect between large and small firms, nor between men and women or non‐union and union members. Overall, these results suggest that the estimated treatment effects are relatively stable across firm size, gender and union membership.

### 5.2 Robustness Checks

In this section, we investigate the robustness of our results using several tests. In Column 1 of Table 7, we exclude robot imports valued at lower than EUR 2,500, which has been suggested to be a potential cut‐off criterion by Acemoglu et al. (2023) as a value below which the robot technology is unlikely to alter the production process substantially. This exclusionary criterion pertains to approximately 4% of individuals within our sample.

**TABLE 7:** Difference‐in‐differences: impact of robotization on SL. Robustness checks.

	(1)	(2)	(3)	(4)	(5)	(6)
	Excl. Low‐value robots	Excl. Industrial real estate spikes	Excl. Import spikes	Excl. Low‐value robots and spikes	Exclude multiple imports	Randomization test
Robotization	1.525	1.696^***^	1.713^***^	1.670^***^	2.078***	0.0654
	(0.307)	(0.309)	(0.312)	(0.325)	(0.386)	(0.420)
Constant	10.16^***^	10.42^***^	10.16^***^	10.03	10.40***	3.451
	(0.392)	(0.385)	(0.394)	(0.391)	(0.547)	(5.070)
Year	Yes	Yes	Yes	Yes	Yes	Yes
Year* subgroup	Yes	Yes	Yes	Yes	Yes	Yes
Entropy balanced	Yes	Yes	Yes	Yes	Yes	Yes
*R* ^2^	0.305	0.308	0.312	0.311	0.299	0.329
*N*	463,236	451,402	456,273	403,717	283,413	365,184

*Note:* Dependent variable: SL days. Standard errors are clustered at the firm level, and the sample is weighted using entropy balance weights. Standard errors in parentheses.

* *p* < 0.05.

**p < 0.01.

*** *p* < 0.001.

A further potential threat to our identification could be that the decision to introduce industrial robots at the firm level is combined with other major investments that affect worker conditions and, in turn, impact SL. In Table 7, Columns 2 and 3, we check to see if our results are robust regarding excluding firms that made other significant investments around the time of robotization. Column 2 excludes firms that exhibited an investment spike in the procurement of buildings and facilities during the year of and/or 1 year prior to robotization,^17^ as a potential worry is that investments to upgrade existing or build new manufacturing sites could potentially be confounded with the effect of robotization if these decisions are taken together. This exclusionary measure affects 5% of the individuals in our dataset.

Given that our measure of robotization is based on imports, we also test to confirm that we are not capturing the combined effect of the imports of other intermediary capital goods in addition to robots on SL. In Table 7, Column 3, we exclude firms that had a spike in their goods imports (excluding industrial robots) in the year of and/or 1 year prior to robotization. In our sample, very few firms exhibited an import spike that was three times the average value of imports, as defined in Bessen et al. (2023). Therefore, we identify firms where, in the year of robotization (and/or 1 year before), imports were 25% higher in monetary value than the average amount for the whole period. This criterion leads to the exclusion of 3% of firms from our analysis. Moving onto Column 4, we exclude firms with a low robot import value as well as those that experienced a spike in industrial real estate and import activity (approximately 14% of firms).

Our identifying assumption is that robotization is a one‐time disruptive event in our difference‐in‐differences event study design. This event is identified as the first instance when a company within our sample imports an industrial robot. This is supported by the fact that a majority (69%) of the firms in our sample follow this pattern and that 77% percent of the total value of industrial robots are imported in the first period.^18^ To check that our results are not driven by firms with multiple import years we exclude them in specification 5. In this specification the effect of robotization on sick leave is even higher and remains statistically significant suggesting that our results are not driven by firms that import multiple times. In all five of these robustness checks, the main result of a positive and statistically significant impact of robotization on the number of SL days remains robust after the exclusion of the observations outlined above. This suggests that our findings are not influenced by low‐value robot imports, plant expansion or an increase in capital stock due to the importation of non‐robot items.

Finally, to check if our results are driven by unobserved changes in SL trends not caused by robotization, we estimate our main specification for a dataset where the timing of robotization is randomly distributed for firms that actually robotized (Table 7, Column 6). We find no significant effects for our randomized year of robotization.

### 5.3 Firm‐Level Analysis

So far, our analysis has focused on individuals who worked at robotizing firms for 3 years before and 4 years after the robotization event. Studies looking at labor market effects of automation and robotization have found that this type of technological change can affect employment (see, e.g., Bessen et al. 2023). Therefore, to investigate the role of compositional effects on the relationship between robotization and SL, in this section, we investigate firm‐level effects with average SL days as the outcome variable of interest. In this specification we lift the restriction that individuals must work at the firm for four consecutive years after the event, allowing for firm‐level compositional changes after robotization. ^19^

**FIGURE 6:**
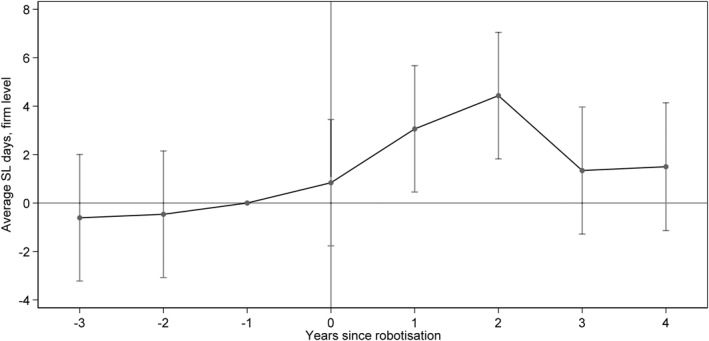
Firm‐level analysis. Difference‐in‐differences event study: impact of robotization on SL days.

Our firm‐level analysis shows an increase in the average firm‐level SL days following robotization, with an increase of around two SL days for the post‐period (Figure 6). The magnitude of the effect of an increase in SL days for the first 3 years following robotization is higher at the firm level than at the individual level of analysis, with an increase of around 2.5 SL days compared to 1.7, respectively (Table 8 compared to Table 3).^20^ Looking more closely at the separation rates for workers with prior sickness spells suggests that employees with a history of sick leave were less likely to leave their jobs post‐robotization, although this effect was less pronounced in the later years following robotization (Figure A10 in the Appendix).

**TABLE 8:** Firm‐level analysis. Difference‐in‐differences: impact of robotization on SL by categories of diagnoses.

	(1)	(2)	(3)	(4)
	All	Muscle	Psychological	Injuries
Robotization	2.490^***^	1.118^*^	0.631	0.235
	(0.636)	(0.442)	(0.191)	(0.157)
Constant	11.45	5.946^***^	1.063^***^	1.074^***^
	(0.837)	(0.582)	(0.252)	(0.207)
Year effects	Yes	Yes	Yes	Yes
*R* ^2^	0.417	0.422	0.316	0.184
*N*	2391	2391	2391	2391

*Note:* Dependent variable: Average SL days at firm. Standard errors in parentheses.

* *p* < 0.05.

**p < 0.01.

*** *p* < 0.001.

Comparing the results of the individual‐ and firm‐level analysis by SL diagnosis shows that robotization caused a higher average increase in SL days for both musculoskeletal and psychological diagnoses at the firm compared to the individual level. Analysis of separations for those with prior psychological diagnoses shows that individuals with this diagnosis are also less likely to leave the firm (Figure A10, panel B, in the Appendix). However, the effect of robotization on injury diagnoses, while positive, is not statistically significant when analyzed at the firm level, while it is for the individual‐level analysis.

## 6 Discussion and Conclusions

In this paper, we study the broader impact of robots on the labor market by looking specifically at worker health. We examine the relationship between robotization and SL among workers in the manufacturing sector in Norway using firm‐level data on imports of industrial robots linked to employee‐firm data. By using a stacked difference‐in‐differences event‐study methodology, we analyze the effect of robotization on the incidence and duration of SL over 4 years following the automation event, while using a control group of firms that robotized later.

Our findings indicate that robotization had a significant effect on SL days, resulting in an increase of approximately 1.7 SL days per year caused by the introduction of industrial robots. As the employer is required to cover lost earnings in the initial weeks of a sickness absence, this corresponds to a significant cost to employers. This is in addition to the cost to taxpayers, who cover lost income for individuals with longer SL spells. The health impact of robotization does not, however, appear to be spread evenly among the workforce, with some occupations more negatively affected than others. Blue‐collar workers are more negatively affected than white‐collar workers, and among the former group, those working on routine tasks are especially vulnerable to SL resulting from robotization. Conversely, robotization does not have a statistically significant effect on any type of SL for *office workers*.

**FIGURE A8:**
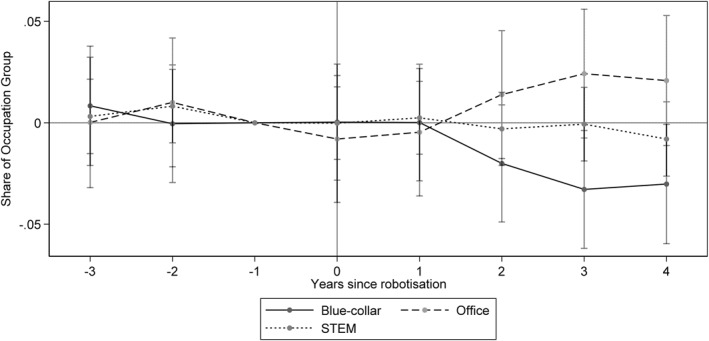
Firm‐level analysis. Difference‐in‐differences event study: share of workers in occupation group.

**FIGURE A10:**
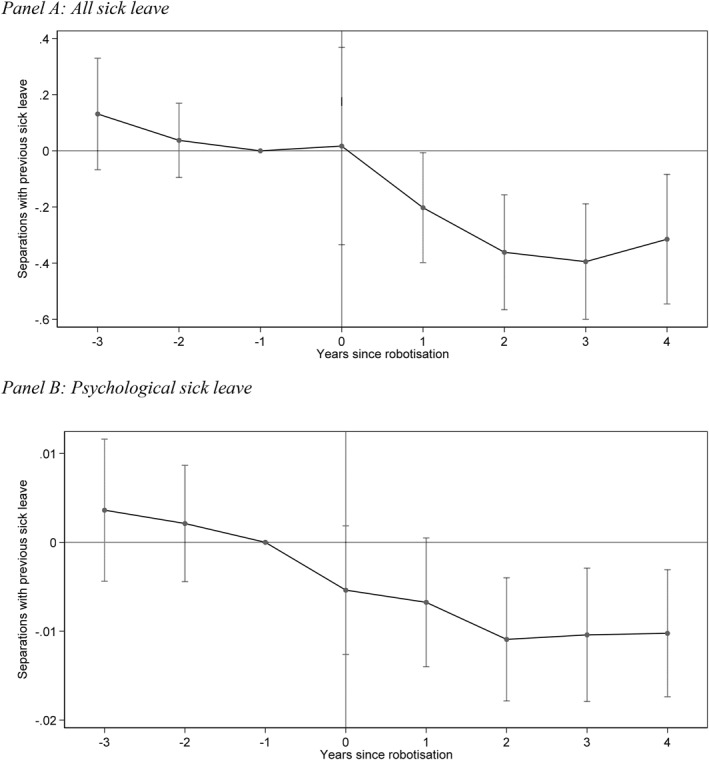
Firm‐level analysis. Difference‐in‐differences event study: impact of robotization on separations with previous sick leave. The above figures show the effects of robotization on the number of job separations at the firm level among those with previous sick leave. The figures show that employees with a history of sick leave were less likely to leave their jobs post‐robotization, although this effect was less pronounced in the later years following robotization.

The effect of robotization on SL varies by diagnosis category. For the workforce taken as a whole, robotization has the largest effect on musculoskeletal diagnoses. Looking at heterogeneity in terms of both occupation groups and diagnoses, we find that robotization increases *blue‐collar* workers' musculoskeletal SL diagnoses most. Firm level analysis shows a reduction in the share of blue‐collar workers following robotization (Figure A8), this could result in a greater work burden on incumbent workers resulting in musculoskeletal ailments. Regarding SL due to injury, we find that only *STEM* workers are negatively affected. This can be explained by the fact that workers in some of these *STEM* occupations are responsible for installing, repairing and maintaining this new technology: tasks that have been shown to involve increased risk of injury. While our individual‐level analysis, which only includes workers who have worked in the firm 3 years before and 4 years after robotization, does not point to any statistically significant effects on psychological SL, our firm‐level analysis reveals a negative health effect. Further investigation into firm separations show that selection plays a role in explaining this difference, as individuals with previous SL spells are less likely to leave their jobs at the robotizing firm in the post‐period.

While there has not been a consensus on the effects of robotization on worker health in the existing literature, this study provides robust results that robotization leads to higher SL both at the individual and firm level in the short to medium term. Our study contributes with micro‐level evidence, providing the missing link between the robotizing firm and the exposed workers in previous studies. Our study primarily provides short‐to medium term effects of robotization on sick leave, but it is important to bear in mind that illness and injury may have long‐term consequences for both individuals and society if this leads to higher rates of disability or reliance on other types of health‐related benefits in the future. While Norway's overall level of robot integration may be lower than other countries, the industries affected have a significant share of robot penetration making our findings relevant for similar industries in other countries.

The finding that robotization may be detrimental to the health of workers, particularly among blue‐collar workers and those in occupations characterized by routine tasks, carries important policy implications. One possible policy intervention is to invest in the development and provision of training programmes for workers, particularly those in occupations that are likely to be impacted by robotization. Such programmes could reduce musculoskeletal health issues by improving job processes associated with new tasks following robotization. Improved safety courses specifically targeted at *STEM* workers who have direct contact with industrial robots could reduce SL related to injuries. In doing so, workers could counter the negative health effects associated with robotization to ensure a smoother transition to a more technology‐driven labor market.

## Conflicts of Interest

The authors declare no conflicts of interest.

## Figures and Tables

**FIGURE A1:**
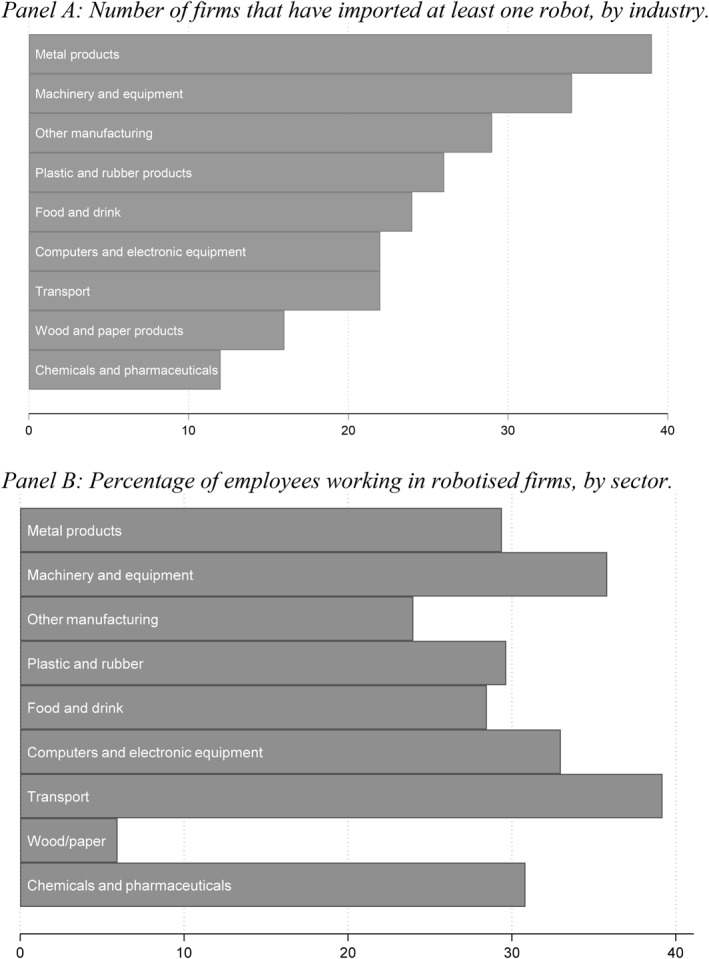
Robot penetration by sector. The figure shows the extent of robot penetration by sector. It includes the top 9 sectors that imported the most robots between 2000 and 2018. Panel A shows **the number** of firms in each industry that have imported at least one robot in the analysis period. Panel B shows the **percentage of employees** in each of these sectors that worked in robotized firms. The categories are based on 2‐digit NACE codes (metal products = 24, 25; machinery and equipment = 28; other manufacturing = 23, 31, 32; plastic and rubber = 22; food and drink = 10, 11, 12; computers and electronic equipment = 26, 27; transport = 29, 30; wood and paper products = 16, 17, 18; chemicals and pharmaceuticals = 19, 20, 21).

**FIGURE A3:**
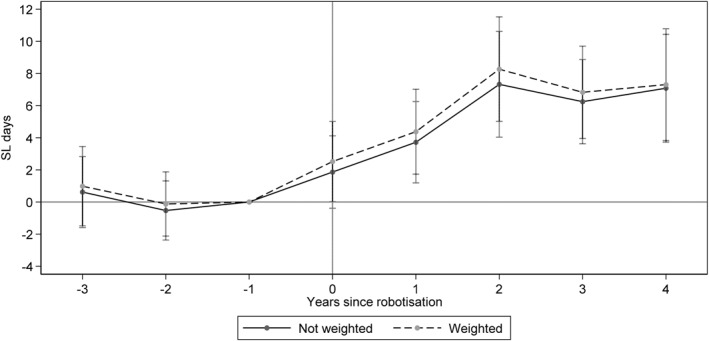
Robotization and SL days for routine workers. Matched and unmatched.

**FIGURE A4:**
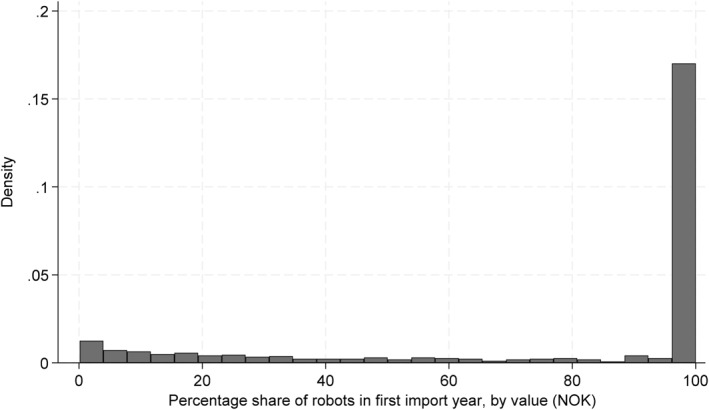
Distribution density of percentage share of industrial robots imported in first year, by value (NOK). Our data includes observations in the years between 2003–2018. We therefore do not know if firms imported robots prior to 2003.

**FIGURE A5:**
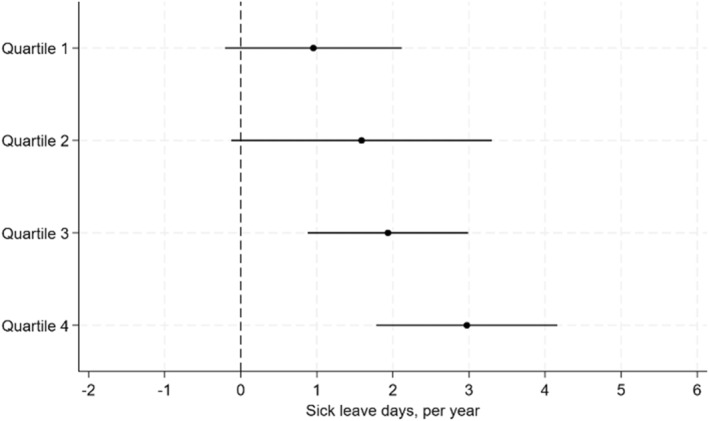
Average treatment effect on SL days, by routineness quartiles. The Figure above shows the average treatment effect for the four post years of robotization on SL. The results are shown by occupational routines measured using O*NET data, where for example quartile 1 represents the 25% least routine occupation and quartile 4 the 25% most routine occupations in our data. Standard errors are clustered at the firm level, and the sample is weighted using entropy balance weights. The whiskers reflect 95% confidence intervals.

**FIGURE A6:**
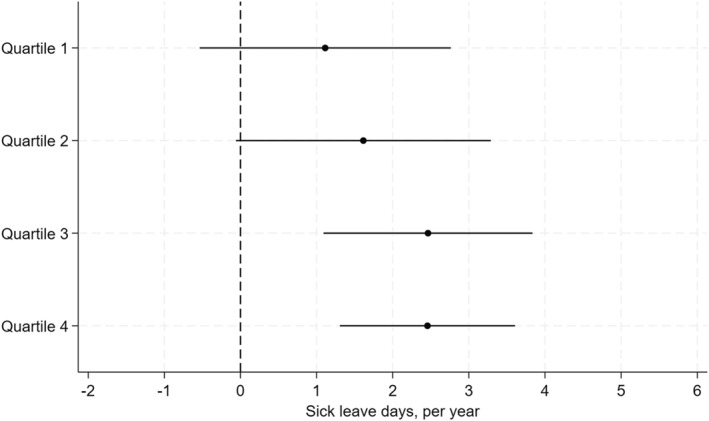
Average treatment effect on SL days, by robot exposure quartiles. The Figure above shows the average treatment effect for the four post years of robotization on SL. The results are shown by robot exposure of firms as defined in Section 5, where for example quartile 1 represents workers in firms with 25% lowest robot exposure and quartile 4 the 25% highest robot exposure. Standard errors are clustered at the firm level, and the sample is weighted using entropy balance weights. The whiskers reflect 95% confidence intervals.

**FIGURE A7:**
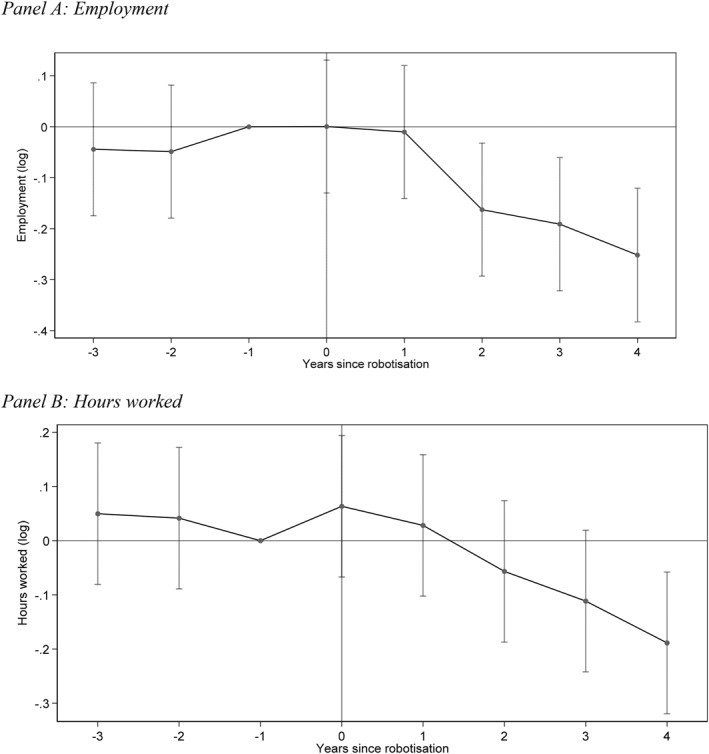
Firm‐level analysis. Difference‐in‐differences event study: impact of robotization on employment and hours worked.

**FIGURE A9:**
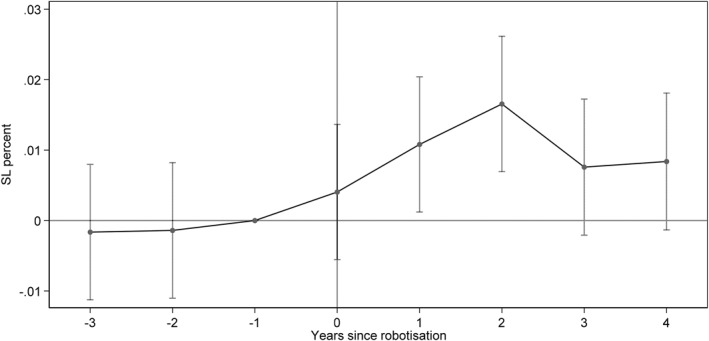
Firm‐level analysis. Difference‐in‐differences event study: impact of robotization on SL percent.

## Data Availability

This paper presents results based on data drawn from Norwegian administrative registers. Due to data confidentiality requirements, we are unable to upload the datasets. Researchers can gain access to the data by submitting a written application to the data owners. Applications must enclose Data Protecting Impact Assessment (DPIA) which should be approved by a data protection officer. Conditional on this approval, Statistics Norway will then determine which data one may obtain in accordance with the research plan. Inquiries about access to data from Statistics Norway should be addressed to: mikrodata@ssb.no. More information is available at: https://www.ssb.no/en/omssb/tjenester‐og‐verktoy/data‐til‐forskning.

## Endnotes

^1^
This approach has been used in the automation literature by Bessen et al. (2023) and Barth et al. (2025), among others.

^2^
Earnings exceeding an upper limit of 6 G, where G is the basic amount in the Norwegian social security system, will not be covered by the social insurance but may be covered by the employer.

^3^
More precisely, we identify firms that have imported the item with the Harmonized System (HS) code 84795000, denoting industrial robots in the import register.

^4^
This discrepancy can be largely attributed to the absence of large‐scale automotive assembly production in Norway.

^5^
In Figure A1 in the appendix we provide alternative measures of robotization intensity which show that while the total number of industrial robots is relatively low, a significant proportion of employees work in robotized firms in the manufacturing sector.

^6^
The ISCO occupation codes that are included in the four categories are shown in Table A2 in the appendix.

^7^
These occupations are in the STEM occupation category.

^8^
Typical occupations in the blue‐collar category in our sample are “assemblers” and “fabricators”. “Service engineer” and “mechanical engineering technician” are common occupations in our STEM occupation category.

^9^
Appendix B outlines how we identify routine and non‐routine workers.

^10^
We have chosen to use later adopters as the control group instead of never‐treated firms due to significant differences in observed and unobserved characteristics between treated and never‐treated firms. Treated firms are typically more high‐tech, larger, and have different worker compositions, making never‐treated firms less suitable as controls. The differences between never‐treated and treated firms are greater than those between early and later‐treated cohorts, which could introduce more bias. This is supported by the close match between our treatment and control groups (Table A3) and parallel pre‐treatment trends (Figure A3).

^11^
A formal parallel trend test for differences between the treatment and control groups in the pre period (*ptrends* in STATA) gives a *p* value of 0.68. As the *p* value is not < 0.05, we fail to reject the null hypothesis, which indicates that the parallel trend assumption is satisfied.

^12^

*Office workers* are combined with STEM ones for the white‐collar occupation category.

^13^
For further insight, we present an analysis in the appendix (Figure A5) where the standardized measure of routineness is divided into quartiles. This analysis reveals that the effect of robotization on sick leave increases linearly with the routineness of tasks.

^14^
Table 2, Column 4 shows the average SL days by diagnosis for our sample of individuals in the pre‐period.

^15^
To provide a more comprehensive analysis of the relationship between robot exposure and its connection to SL, we have added a figure in the appendix (Figure A6) that illustrates the average treatment effect across the four quartiles of robot exposure.

^16^

*Office workers* have significantly lower unionization rates than blue‐collar and STEM workers and, as shown above, are less affected by robotization when it comes to health outcomes. In our sample, blue‐collar, STEM and *office workers* have unionization rates of 83%, 67% and 49%, respectively.

^17^
Our definition of an investment spike follows the approch used by Bessen et al. (2023).

^18^
We include a distribution density figure of the percentage share of industrial robots imported in the first year, by value (NOK) in Figure A4 in the appendix.

^19^
Using the same specification, we also look at employment and hours worked as outcome variables and find that robotization is negatively associated with employment (around a 20% reduction) at the firm level for our sample. More details are provided in Appendix G, Figures A7 and A8.

^20^
An alternative measure of SL at the firm level is SL percent (total days of SL as a share of the total work days). Our findings are similar when using this measure. The results are provided in Appendix G, Figure A9 and Table A5.

## Appendix A Definition of Variables

**TABLE A1:** ICPC‐2 codes for injuries.^21^

ICPC‐2 codes	Description	ICPC‐2 codes	Description
A80	Trauma/injury NOS^22^	L74	Fracture: Hand/foot bone
A81	Multiple trauma/injuries	L75	Fracture: Femur
A82	Secondary effect of trauma	L76	Fracture: Other
A84	Poisoning by medical agent	L77	Sprain/strain of ankle
A85	Adverse effect medical agent	L78	Sprain/strain of knee
A86	Toxic effect non‐medicinal substance	L79	Sprain/strain of joint NOS^22^
A87	Complication of medical treatment	L80	Dislocation/subluxation
A88	Adverse effect physical factor	L81	Injury musculoskeletal NOS^22^
A89	Effect prosthetic device	N79	Concussion
B76	Ruptured spleen traumatic	N80	Head injury other
B77	Injury blood/lymph/spleen other	N81	Injury nervous system other
D79	Foreign body digestive system	R87	Foreign body nose/larynx/bronchus
D80	Injury digestive system other	R88	Injury respiratory other
F75	Contusion/hemorrhage eye	S12	Insect bite/sting
F76	Foreign body in eye	S13	Animal/human bite
F79	Injury eye other	S14	Burn/scald
H76	Foreign body in ear	S15	Foreign body in skin
H77	Perforation ear drum	S16	Bruise/contusion
H78	Superficial injury of ear	S17	Abrasion/scratch/blister
H79	Ear injury other	S18	Laceration/cut
L72	Fracture: Radius/ulna	S19	Skin injury other
L73	Fracture: Tibia/fibula	U80	Injury urinary tract

^22^ Not otherwise specified.

## Appendix B Occupation Categories

### Occupations

We categorize the occupations of individuals in our sample into (1) *blue‐collar workers* (excluding machine operators), (2) *office workers* and (3) *STEM workers*. The register data that we use contain ISCO‐88 occupation classifications that we place into these four categories based on the task contents of the jobs. As we only focus on individuals that work in robotizing firms, the workers in our sample do not cover all the possible occupations listed in the ISCO‐88 classification system. Examples of such occupations are teaching, agricultural work and the armed forces.

**TABLE A2:** Classification of ISCO‐88 codes into occupation groups.

ISCO‐88 code	Description
Blue‐collar	
7	Craft and related trades workers
8	Plant and machine operators and assemblers
9	Elementary occupations
Office worker	
1	Managers
2 (excl. 21)	Professionals (excluding engineering science professionals)
3 (excl. 31and 32)	Associate professionals (excluding engineers and technicians)
4	Office clerks/secretaries/customer service, etc.
5	Service workers
STEM	
31	Physical and engineering science associate professionals
32	Other technical professions (lab technicians, product developers etc.)
21	Physical, mathematical and engineering science professionals

*Note:* The codes refer to ISCO‐88; see https://ec.europa.eu/eurostat/documents/1978984/6037342/ISCO‐88‐COM.pdf for a full list of four‐digit codes.

### Routine and Non‐Routine Tasks

We used O*NET data to create indicators for the extent to which each occupation contains tasks that can be categorized as routine. We used crosswalks from the National Crosswalk Service Center to ensure that the occupational categories used in the O*NET database were compatible with Norwegian STYRK occupation codes, which are closely related to the International Labor Organization's International Standard Classification of Occupations (ISCO).^22^ We follow Hummels et al. (2014) to identify components that typify routine tasks. The routine task variable is composed of data from the components of manual dexterity, finger dexterity, multi‐limb coordination, processing information and evaluating information to determine compliance with standards. We categorize individuals who have one or more standard deviations above the mean level of routineness among all occupations in Norway as being routine workers.

## Appendix D Entropy Balancing

As well as excluding never treated individuals, an additional step to ensure comparability between the treatment and control groups, we employ the entropy balancing procedure introduced by Hainmueller (2012). This method, a non‐parametric data pre‐processing approach developed for binary treatment studies, is designed to achieve balance in the means and higher moments of specified variables between the treatment and control groups. This approach not only addresses the issue of orphan observations but also recognizes that outlier data points most likely have limited relevance to the analysis and assigns them reduced weights.

We use entropy balancing to weight the observations in the control group to match the characteristics of the treatment group along the dimensions of wages, age, gender, education and company size. As we used data that are stacked by cohorts, we conduct the entropy balancing procedure for each cohort separately. Panel A in Table A3 shows the unweighted descriptive statistics for the treatment and control groups in our data. It shows that the age, gender and education balance is similar between the treatment and control groups. There are, however, somewhat more substantial differences in workers' wages and the size of firms (number of employees) between the treatment and control groups. Consequently, entropy balancing has the largest effect on these two variables. Panel B shows the summary statistics for the weighted variables, where wages and employment are more similar between the control and treatment groups relative to the unweighted sample.

**TABLE A3:** Individual characteristics treatment and control entropy balance weighting.

Panel A: Non‐weighted						
	Treatment			Control		
	Mean	Variance	Skewness	Mean	Variance	Skewness
Wage	575,975	2.60 E + 11	46.06	510,534	6.32 E + 10	10.00
Age	43.52	101.47	−0.01	42.52	81.30	−0.12
Female	0.20	0.16	1.53	0.25	0.19	1.15
University	0.29	0.20	0.95	0.26	0.19	1.11
Employees	985	1,575,205	4.99	1556	4,223,545	2.15

Panel B: weighted						
	Treatment			Control		
	Mean	Variance	Skewness	Mean	Variance	Skewness
Wage	576,536	2.61 E + 11	45.98	576,437	1.04 E + 11	11.00
Age	43.53	101.53	−0.01	43.53	82.53	−0.21
Female	0.20	0.16	1.53	0.20	0.16	1.53
University	0.29	0.20	0.94	0.29	0.20	0.95
Employees	985	1,574,099	5.00	950	5,246,881	9.06

An example of the impact of entropy balancing on our estimates is shown in Figure A3. It compares the results of the stacked difference‐in‐differences estimation for routine workers with and without entropy balancing weights. Using individuals who worked in firms that robotized later already provides a good control group, and the balancing procedure improves the comparability of the treatment and control groups further.

## Appendix F

**TABLE A4:** Difference‐in‐differences: Impact of robotization on taking any sick leave by categories of diagnoses.

	(1)	(2)	(3)	(4)
	All	Muscle	Psychological	Injuries
Robotization	0.00102	0.00369	0.00105	0.00390^*^
	(0.00413)	(0.00293)	(0.00165)	(0.00181)
Constant	0.354	0.165^***^	0.0399	0.0570^***^
	(0.00614)	(0.00455)	(0.00242)	(0.00253)
Year effects	Yes	Yes	Yes	Yes
Entropy balanced	Yes	Yes	Yes	Yes
*R* ^2^	0.374	0.324	0.247	0.201
*N*	473,041	473,041	473,041	473,041

*Note:* The above table shows the stacked difference‐in‐differences two‐period estimation results, comparable to Table 3. Standard errors in parentheses.

* *p* < 0.05.

***p* < 0.01.

*** *p* < 0.001.

## Appendix G Robotization and Employment. Firm‐Level Analysis

**TABLE A5:** Firm‐level analysis.

	(1)	(2)	(3)	(4)
	All	Muscle	Psychological	Injuries
Robotization	0.0102***	0.00409*	0.00255	0.000867^a^
	(0.00234)	(0.00185)	(0.000613)	(0.000495)
Constant	0.0368***	0.0194***	0.00319	0.00305***
	(0.00308)	(0.00243)	(0.000806)	(0.000651)
Year effects	Yes	Yes	Yes	Yes
*R*2	0.473	0.380	0.636	0.181
*N*	2391	2391	2391	2391

*Note:* Dependent variable: Sick leave percent. Standard errors in parentheses.

^a^ *p* < 0.10.

* *p* < 0.05.

** *p* < 0.01.

*** *p* < 0.001.
